# [Expression of Concern] Simvastatin potentiates doxorubicin activity against MCF-7 breast cancer cells

**DOI:** 10.3892/ol.2026.15835

**Published:** 2026-08-27

**Authors:** Benjaporn Buranrat, Wanwisa Suwannaloet, Jarinyaporn Naowaboot

Oncol Lett 14: 6243–6250, 2017; DOI: 10.3892/ol.2017.6783

Following the publication of the above article, a concerned author has drawn the authors’ attention to the fact that the caspase-3 enzymatic activity reported in Fig. 3 on p. 6247, and the caspase-3 western blotting data shown in Fig. 4 on p. 6248, were surprising results given the use of the MCF-7 cell line in this study. These findings relating to changes in caspase-3 activity or expression were not anticipated, since it has been demonstrated that MCF-7 cells do not express caspase-3 [for example, see the paper by Jänicke and colleagues entitled “MCF-7 breast carcinoma cells do not express caspase-3.” Breast Cancer Res. Treat., vol. 117.1 (2009); p. 219–221].

The authors have been contacted by the Editorial Office to offer an explanation for these unexpected findings in this paper, and we are awaiting their response. Owing to the fact that the Editorial Office has been made aware of potential issues surrounding the scientific integrity of this paper, we are issuing an Expression of Concern to notify readers of this potential problem while the Editorial Office continues to investigate this matter further.

